# Dysmetabolisms Can Affect Total Antioxidant Capacity (TAC) of Human Plasma: Determination of Reference Intervals of TAC by Way of CUPRAC-BCS Method

**DOI:** 10.3390/antiox10010058

**Published:** 2021-01-05

**Authors:** Enrico Prenesti, Silvia Berto, Fabio Gosmaro, Marco Bagnati, Giorgio Bellomo

**Affiliations:** 1 Department of Chemistry, University of Turin, Via Pietro Giuria 5, 10125 Turin, Italy; enrico.prenesti@unito.it (E.P.); fabiogos@yahoo.it (F.G.); 2 Istituto Professionale di Stato per Servizi Alberghieri e Ristorazione G. Giolitti, Piazza IV Novembre, 12080 Mondovì (CN), Italy; 3 Major of the Charity Hospital, University of Eastern Piedmont Amedeo Avogadro, C.so Giuseppe Mazzini, 18, 28100 Novara, Italy; marco.bagnati@maggioreosp.novara.it (M.B.); bellomo.giorgio@gmail.com (G.B.)

**Keywords:** total antioxidant capacity, reference intervals, CUPRAC, human plasma, healthy population, dysmetabolism

## Abstract

The total antioxidant capacity (TAC) of human plasma is an index of the redox buffer capacity of this biological fluid and could be a biomarker for those disorders affecting redox status. Distinguishing physiological from pathological conditions needs a reference. Therefore, this work aims to define the reference intervals for TAC of human plasma of apparently healthy adult individuals. TAC was measured using the CUPRAC-BCS (CUPric reducing antioxidant capacity-bathocuproinedisulfonic acid) method previously optimized and tested in a clinical laboratory. A population of 500 blood donors was selected, plus an additional 222 pathological patients carrying specific defective metabolisms, namely, hyperuricemia, hyperbilirubinemia, and type 2 diabetic mellitus. The reference intervals of TAC were calculated according to international guidelines. Due to the response of a partitioning test, the reference intervals for healthy population were separately defined for male (258) and female (151) groups. The reference intervals (µmol L^−1^) resulted: 727–1248 for the male subgroup and 637–1048 for the female subgroup. The absence of an age effect on TAC values was verified. The reference intervals evaluated allow a discussion on some pathological conditions overloading the plasma with redox-active waste substances.

## 1. Introduction

The primary aim of a clinical measurement system is to improve healthcare delivery. Quality must involve instrumentations, materials, methods and practices in a growing coordinated effort of improvement. The primary goal of quality in measurement in the clinical laboratories is creating a transparent and stable system that is embedded into clinical workflows. The environment of the chemical-clinical hospital laboratory in itself represents an excellent challenge for measurement, since there is a need for speed and assiduity in a service to the community centered on the management of the disease or, in any case, on the need for support in diagnosis or therapies. Particularly, clinical chemistry needs appropriate standards, for each biochemical parameter of interest, to deliver criteria for decisions to physicians. Total antioxidant capacity (TAC) is a parameter for which these standards are still missing and whose clinical significance has not yet been properly understood. 

There is recent growing evidence that dietary antioxidants [1] may be associated with a risk reduction of oxidative stress-related diseases. In a paper by La Vecchia et al. [2], dietary TAC was found to be inversely related to colorectal cancer risk (it was the first study indicating consistent inverse relations between dietary TAC and colorectal cancer risk with over 6000 patients examined). Guo-Chao Zhong et al. [3] found that dietary TAC can be inversely associated with pancreatic cancer incidence while Karimi et al. [4] wrote that the risk of breast cancer can be reduced by consuming food with high TAC values. These and other results on the topic stimulate further deepening of the clinical meaning of TAC in view of its routine use.

TAC of body fluids is due to both dietary and metabolic contributions, which are interrelated. Homeostatic processes regulate the local redox conditions determining a balance between pro-oxidant and antioxidant substances favorable to health [1]. The aerobic metabolism physiologically produces reactive oxygen and nitrogen species (ROS, RNS) that require a buffering capacity to avoid molecules and tissue damages caused by their excess [5,6,7].

Physiological antioxidants can be subdivided according to two classification criteria: the first is related to the activity compartment, namely intra- and extracellular, and the second is related to the origin, namely endo- and exogenous. Extra- and intracellular antioxidant buffering capacities are regulated by a multiplicity of biochemical processes involving biomolecules of various chemical natures [8]. Intracellular antioxidants include metalloenzyme (endogenous proteins) localized in the cytoplasm and in specific organelles of each cell. Extracellular antioxidants are a complex blend of molecules coming from both metabolism and diet. Both intra- and extracellular antioxidant molecules are food dependent. In particular, the biosynthesis of intracellular enzymes depends upon the presence of both essential amino acids and mineral components (namely, Cu, Zn, Mn, Fe and Se), while exogenous extracellular antioxidants include vitamins (organic micronutrients) and polyphenols (bioactive phytochemicals) [9].

The inefficacy of the redox buffer system leads to an imbalance of the redox-active substances that causes oxidative stress, an occurrence that is supposed to be involved in a variety of pathogenic phenomena such as mutation, carcinogenesis, inflammation, aging and signal transduction [7]. The measurement of a chemical parameter correlated to the redox status of a body fluid might therefore give clinicians useful data for managing health.

TAC is an index of redox reactivity of a fluid in fixed experimental conditions and is detected using a redox test reaction. TAC does not represent the concentration of a specific substance, and its value is method dependent. From a biochemical viewpoint, the TAC of human plasma can also be named Non-Enzymatic Antioxidant Capacity (NEAC), as suggested by Bartosz et al. [10], to highlight that the antioxidant capacity of the human plasma does not reflect the antioxidant enzymatic action. In a similar way, from a biological viewpoint, the TAC of human plasma could be also defined as extracellular total antioxidant capacity. In our opinion, any possible misunderstanding disappears specifying (how it should always be). TAC of human plasma and remembering that the adjective total is a consequence of the measurement principle of the TAC, which is based on a chemical reaction test providing a cumulative response caused by a blend of redox-active substances able to react under the selected test conditions.

The TAC can be measured with many different methods [11,12,13,14,15] depending upon the chemical reactions test selected for its detection and quantification. Our research group chose the CUPRAC-BCS (CUPric reducing antioxidant capacity-bathocuproinedisulfonic acid) photometric method since it works at a physiological pH, the measurement procedure is simple and fast, the stability of reagents is satisfactory and it is analytically robust and inexpensive and easily automatized for massive routines [16] and references therein. In our first paper we optimized the working conditions of the method [16], planning and testing it in a hospital clinical laboratory routine and working on a pool of plasma. The measurement uncertainty of the method was then subsequently evaluated [17] to assess its analytical performance.

In this work, we aim to collect biochemical data on apparently healthy individuals of a selected adult population with the view to define the reference intervals for TAC. To test the responsivity of the reference intervals to pathological conditions, the TAC value was measured on subjects carrying widespread dysmetabolisms. Despite the abundance of scientific papers dealing with the redox chemistry of human plasma, a literature search revealed very few studies reporting the reference intervals estimation of TAC. Nevertheless, the antioxidant status of a body fluid can be considered a clinical biomarker only when accompanied by a reference. The paper by Kampa et al. [18] reports an estimation of the reference intervals obtained by way of both crocin bleaching and TEAC (trolox equivalent antioxidant capacity) assays on 44 human blood donors. Habdous et al. [19] estimated the reference intervals measuring the TAC on serum of 463 subjects with the TEAC assay.

According to the International Federation of Clinical Chemistry and Laboratory Medicine (IFCC), the a priori approach [20] was adopted for the selection of reference individuals. In this perspective, 500 blood donors were chosen as a healthy population. We aim to circumstantiate the clinical meaning and use of TAC and, for this reason, reliable reference intervals on healthy subjects are necessary, also in view of a future widening of the study. The clinical meaning of the TAC was further investigated measuring the number of 222 pathological subjects carrying specific defective metabolisms, namely, hyperuricemia (including treatment phases of pre- and post-dialysis), hyperbilirubinemia and type 2 diabetic mellitus.

## 2. Materials and Methods

### 2.1. Chemicals

Cu(II) sulphate pentahydrate (purity ≥ 98%), bathocuproinedisulfonic acid (BCS, purity ≥ 98%), PBS (phosphate buffered saline 0.1 mol L^−1^), L-ascorbic acid (purity ≥ 98%), bilirubin (purity ≥ 96%) and uric acid sodium salt (purity ≥ 98%) were Sigma Aldrich (St. Louis, MO, USA) products. Copper(II) reference solution was by Merck (Darmstadt, Germany) (1000 ± 1) mg L^−1^. ADVIA Chemistry Uric Acid Concentrated Reagent and ADVIA Chemistry Total Bilirubin_2 were purchased from Siemens Healthcare Diagnostics Inc (Tarrytown, NY, USA). Ready to use pH-metric buffer solutions (pH 4.01 and 9.00 at 20 °C) were from Merck (Darmstadt, Germany).

Solutions were prepared in grade A glassware and diluted by ultrapure water (MilliQ quality).

The calibration solutions of copper(II) were prepared by diluting the reference solution up to concentrations ranging between 0.2 and 2.0 mmol L^−1^. BCS was dissolved in 10 mmol L^−1^ PBS (pH 7.40) up to the concentration of 36 mmol L^−1^ (stock solution). Stock solutions of both copper(II) sulphate and L-ascorbic acid were obtained by dissolving the solids in MilliQ water to respectively reach concentrations of 10 mmol L^−1^ and 50 mmol L^−1^. The two solutions (namely R1 and R2) inserted into the reagent trays of the automatic measurement system were: reagent R1, BCS 900 µmol L^−1^ in PBS buffer; reagent R2: (i) copper(II) sulphate 640 µmol L^−1^ in MilliQ water, when used for the sample preparation, or (ii) L-ascorbic acid 10 mmol L^−1^ in MilliQ water, when used for the calibration.

### 2.2. Apparatuses

Clinical chemistry auto-analyzer ADVIA 2400 Chemistry System, provided by Siemens, Munich, Germany, with 2400 test/h capacity and two reagent trays (R1 and R2). Mettler MS204S balance. pH-meter pH-211 Hanna Instruments equipped with a Porotrode pH glass electrode by Metrohm, Herisau, Switzerland was also employed.

### 2.3. Procedures

The visible photometric determinations of uric acid, total bilirubin and total antioxidant capacity, were carried out using ADVIA 2400.

Uric acid was quantified using the ADVIA Chemistry Uric Acid Concentrated Reagent, a commercial kit purchased from Siemens Healthcare Diagnostics Inc (Tarrytown, NY, USA). The analyte is converted by uricase into allantoin and hydrogen peroxide. Due to the catalytic action of the peroxidase, a colored complex is formed by hydrogen peroxide, stoichiometric with uric acid, 4-aminophenazone and N-ethyl-N-(2-hydroxy-3-sulfopropyl)-3-methylaniline. The absorbance is then measured. 

Bilirubin was quantified by the ADVIA Chemistry Total Bilirubin_2 method based on the chemical oxidation of the analyte with vanadate ion to biliverdin.

The description of the procedure used for the TAC quantification was fully described in [16,17]. In this work copper(I) was used as reference species. The calibration curve was obtained by reducing the copper(II) of the reference solutions to copper(I) by L-ascorbic acid. The method is based on the analytical reaction Cu(II)-BCS + S_rid_ → Cu(I)-BCS + S_ox_, where Cu(II)/(I)-BCS are the copper complexes with BCS and S_rid/ox_ indicating the redox-active substances in the sample. The Cu(I)-BCS complex, stoichiometrically formed by the reducing agents of the sample active in the test conditions, was detected by photometric measurement (478 nm).

### 2.4. Subjects

According to the International Federation of Clinical Chemistry and Laboratory Medicine (IFCC), the a priori approach [20] was used for the selection of reference individuals. In this perspective, the blood donor population was chosen as a healthy adult population, in order to exclude: (i) risk factors (obesity, hypertension, anemia), (ii) intake, of pharmacologically active agents (drug treatments for diseases or suffering, drug abuse, ethyl alcohol) and (iii) specific physiological state (pregnancy). Plasma from 500 blood donors was collected. Table 1 shows the conditions for blood donors assumed to be in a good healthy state in order to determine the reference intervals of the TAC parameter according to the CUPRAC-BCS method. After a screening, 413 patients met the established criteria of good health conditions.

The clinical meaning of TAC was further investigated by measuring the TAC of pathological subjects carrying specific defective metabolisms. In particular, the investigation was focused on diseases overproducing redox-active molecules able to significantly affect the TAC value. Plasma was collected from: (i) 54 hyperuricemic patients (males); (ii) 25 hyperbilirubinemic patients (males); (iii) 50 dialysis patients (50 pre-dialysis blood samples and 50 post-dialysis blood samples) and (iv) 93 type 2 diabetic mellitus patients therapized (55 males and 38 females).

In this study they were simply used leftovers of plasma samples, accompanied with anonymized data. Plasma samples were always collected during routine sessions, scheduled for purposes unrelated to the study. The collection of experimental data was carried out to increase knowledge of the redox chemistry of plasma, without diagnostic or therapeutic goals.

### 2.5. Plasma Sample Collection and Preparation

Blood from apparently healthy (blood donors) and pathological subjects—hyperuricemic, hyperbilirubinemic, dialysis and diabetic patients—was gathered into lithium-heparin containing tubes. The blood was centrifuged at 3500 rpm for 6 min at 15 °C obtaining plasma as supernatant.

### 2.6. Statistical Treatment of Reference Intervals

The data treatment included the partitioning of the reference intervals into appropriate groups and the inspection of the distribution of each group, the identification of outliers and the determination of reference intervals.

The variation among subgroups was investigated according to the CLSI—Clinical Laboratory Standards Institute [21]. The Harris and Boyd’s test for partitioning of reference intervals [22] was used. This test assumes Gaussian distribution of the subgroups and it is based on the following criterion:

(1) (x¯a−x¯b)(sa2na+sb2nb)12≥5((na+nb)/2120)12

where:

- *s_a_* is the standard deviation of subgroup *a*,
- *s_b_* is the standard deviation of subgroup *b*,
- *n_a_* is the number of subjects of subgroup *a*,
- *n_b_* is the number of subjects of subgroup *b*,
- ${\bar{x}}_{a}$ is the mean of subgroup *a*,
- ${\bar{x}}_{b}$ is the mean of subgroup *b*.

If the above criterion is verified it is necessary to estimate reference intervals for both subgroups separately. The requirement of partitioning the reference intervals into male and female subgroups was tested. D’Agostino–Pearson [23] test was used to verify the normality of distributions of each subgroup.

The identification of outliers in the dataset was performed as described by Tukey [24] and the outliers were removed from the original dataset, according to Horn et al. [25], before going on with the determination of the reference intervals.

According to IFCC [26], the nonparametric method, in its bootstrap version, for determination of reference intervals was used. Particularly, this method makes no assumptions concerning the type of distribution and does not use estimation of distribution parameters. The reference intervals are defined as the central 95% of the measurements. The lower reference limit is the 2.5th percentile while the upper reference limit constituted the 97.5th percentile for the population. The percentiles are determined simply by cutting off the required percentage of values in each tail of the subset reference distribution. As a rule, when estimation of the reference intervals is performed in its bootstrap version, the results obtained are preferable to those provided by the parametric method.

The bootstrap estimation of reference intervals [22,27,28] consists of the following steps: (i) draw, with replacement, random samples of size n from the subset of n raw data. The number of resampling is settled to 1000; (ii) for each resample, the upper and lower reference limit (i.e., the 2.5th and 97.5th percentiles) are estimated and saved; (iii) upon completion of all iterations, the median of the resample estimates of each of the two reference limits is used as final estimates; and (iv) finally, the 0.90 confidence interval (i.e., the 5th and 95th percentiles) of each reference limit is performed to obtain an estimation of variability.

### 2.7. Investigation on Age Effect

Each sex-based subgroup was further investigated in order to investigate the relationship between TAC and age applying a one-way ANOVA test. The subgroups were subdivided into four age-intervals, particularly: 18–30 y; 31–40 y; 41–50 y and 51–65 y.

### 2.8. Software

Origin 6.1. (by OriginLab Corporation, Northampton, MA, USA) and SPSS Statistics 17.0 (by SPSS, Segrate, MI, Italy) were used for data processing and presentation. R software (created by Ross Ihaka and Robert Gentleman at the University of Auckland, Auckland, New Zealand, and now developed by the R Development Core Team; it is freely available under the GNU General Public License) was used for bootstrap estimation of the reference values.

## 3. Results

### 3.1. Preliminary Statistical Treatment

The inspection of the distribution of male and female groups was performed through the D’Agostino–Pearson test and the results are collected in Table S1 in the Supplementary Materials. It was possible to confirm the Gaussian distribution of the two subgroups. The outliers of the two datasets were identified and removed (Figures S1 and S2, and Table S2). Thus, Harris and Boyd’s test for partitioning of reference intervals was performed on the two new subgroups (Table S2: males—258; females—151). The criterion of Equation (1) was verified, and partitioning was recommended. Therefore, the reference intervals were separately defined for the male and female groups.

### 3.2. TAC Reference Intervals

The reference intervals for male and female groups were determined with the procedure described before on a dataset of 409 individuals, 258 males and 151 females. The reference interval of the male subgroup resulted in being equal to 727–1248 µmol L^−1^, with the 0.90 confidence interval of 690–778 µmol L^−1^, for the lower limit, and 1223–1302 µmol L^−1^, for the upper limit, while for the female subgroup 637–1048 µmol L^−1^, with the 0.90 confidence interval of 608–681 µmol L^−1^, for the lower limit, and 1037–1134 µmol L^−1^ for the upper limit. Results are listed in Table 2.

Kampa et al. [18] reported an estimation of the reference intervals obtained by way of both crocin bleaching and TEAC assays on 44 human blood donors. The reference values of TAC were estimated to be 1.175 ± 0.007 mmol L^−1^ (crocin bleaching assay) and 1.209 ± 0.005 mmol L^−1^ (TEAC assay). According to the authors’ explanation, their results are achieved and expressed as mean ± standard error mean. This mode of estimation is noncompliant with the guidelines by IFCC and CLSI and a critical comparison seems inappropriate and not applicable with the finding of this work. On the other hand, Habdous et al.’s [19] estimation of the reference intervals of TAC (TEAC assay; results expressed as TAS, total antioxidant status, instead of TAC) is compliant with the IFCC guidelines. They reported values of TAC as mean ± standard deviation varying from 1.56 ± 0.11 mmol L^−1^ in women (163 subjects; range age 20–65 years) to 1.67 ± 0.13 mmol L^−1^ in men (160 subjects; range age 20–65 years) with an overall range varying from 1.18 to 2.11 mmol L^−1^ after test for normality (*p* > 0.05). The statistical treatment revealed the necessity of partitioning the individuals into male and female subgroups, in agreement with our findings.

### 3.3. Age Effect

**Figure 1:**
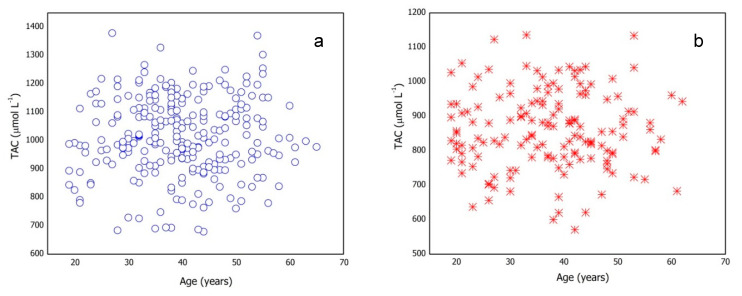
Total antioxidant capacity (TAC) values vs. age for (**a**) male and (**b**) female subgroups.

The effect of age on the TAC value was also investigated. Figure 1 shows the trend of the TAC values vs. age for male and female subgroups previously selected for the reference intervals determination. No specific trend was observed between the quantities under consideration.

The subgroups were subdivided in four age-intervals (Table S3) and one-way ANOVA was applied to investigate the relationship between TAC and age. Table S3 reports statistical parameters for each age-interval and one-way ANOVA results. The results show that age does not significantly affect TAC values.

This finding agrees with data reported by Habdous et al. [19]. The presence of age-based subgroups inside male and female groups is statistically unsupported.

### 3.4. TAC for Pathological Patients

**Figure 2:**
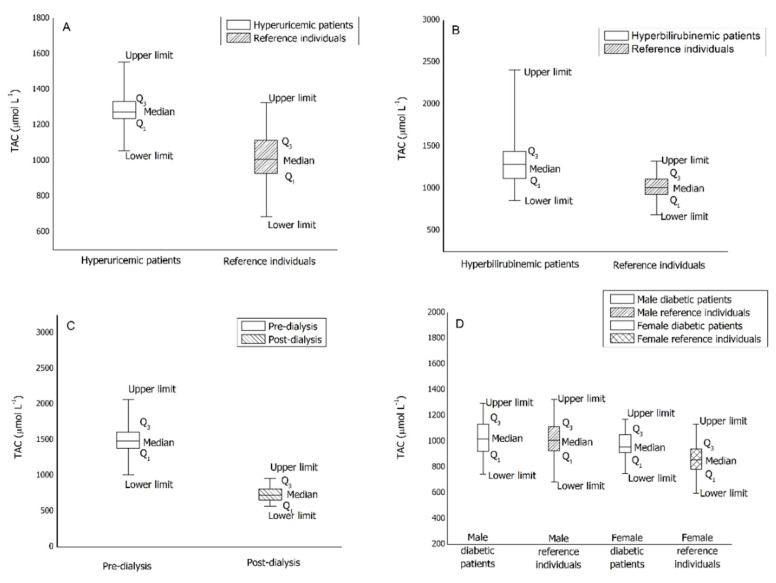
Box-whiskers plots of TAC values for pathological patients. (**A**): hyperuricemic patients; (**B**): hyperbilirubinemic patients; (**C**): dialysis patients; (**D**): diabetic patients.

Table S2 displays the descriptive statistic for the four pathological groups under study. The comparisons of the median values reported in Table S2 with the reference intervals are shown in Figure 2 as a box-whiskers plot.

From Figure 2A, it is evident that the median of the TAC value for hyperuricemic male patients is in the upper limit zone of the reference intervals. Moreover, the range including 25th and 75th percentiles results significantly narrowed compared to that of healthy patients.

From Figure 2B, it is evident that the median of the TAC value for hyperbilirubinemic male patients is in the upper limit zone of the reference intervals and the pathological group shows an extraordinarily high upper limit. Moreover, the range including 25th and 75th percentiles results significantly widens compared to that of healthy patients. 

Plasma from 50 dialysis patients—50 pre-dialysis blood samples and 50 post-dialysis blood samples—was collected and the TAC determined. The values reported in Table S2 and the Figure 2C clearly show that the dialysis treatment induces a significant reduction of TAC values. Moreover, by comparing the median value for the pre-dialysis plasma samples with the reference intervals, it is evident that the median of the TAC values for dialysis patients exceeds both males’ and females’ reference limits. On the other hand, the median value for the post-dialysis samples resulted in the lower limit zone of the reference intervals. In addition, pre-dialysis TAC values are quite dispersed while post-dialysis ones are more compacted, as is typical of a forced clinical treatment.

Plasma from 93 diabetic patients (type 2 diabetes mellitus therapized)—55 males and 38 females—was collected and the TAC determined. Comparing the median values reported in Table S2 with that of the reference intervals previously obtained, it is evident that the median of the TAC values for diabetic patients is included inside the reference intervals for both the male and female groups. Figure 2D shows no significant differences in term of distribution; therefore, type 2 diabetes mellitus seems to have no effect on the TAC value.

## 4. Discussion

The CUPRAC-BCS method turned out very suitable for a routine measurement of TAC of human plasma. It is analytically robust, and it can measure TAC values included in a wide range, enabling the TAC measurement of both healthy and pathological patients. 

The reference intervals, evaluated according to the IFCC for both men and women, allow for a discussion of those pathological conditions overloading the plasma with redox-active waste substances. In the literature, Cao et al. [29] stated that an increase of antioxidant capacity of plasma is a consequence of absorption of antioxidants after consumption of a diet high in fruit and vegetables. The results of our work, in accordance with the findings of Notas et al. [30], show that an increase of TAC of plasma is not necessarily a consequence of a large intake of dietary antioxidants but could also be due to a metabolic alteration. In particular, the ineffectiveness of an internal organ which causes accumulation of a redox-active substance in the human plasma—such as uric acid, due to renal failure, or bilirubin, due to icteric status (both derived from nitrogen catabolism)—leads to the overcoming of the TAC upper limits estimated for the healthy population. On the other hand, the contribution of uric acid to the TAC of plasma is well known. [31,32,33]. 

It should be highlighted that the meaning of the TAC value depends upon the nature of the sample under study. For example, a food sample is considered healthier the higher the TAC value, while for a biological sample the desired healthy conditions correspond to a TAC value falling into a specific range. Such a range can be exceeded, since plasma is an extracellular fluid and collects molecules deriving from metabolic processes being excreted. The evaluation of the TAC reference intervals is then the crucial step to attain its clinical applications aimed to distinguish physiological from altered conditions. 

The results achieved in this work allow delimiting the field of disorders able to affect the redox chemistry of plasma measured by TAC. TAC can be a biomarker for those disorders affecting the redox status of body fluids, having defined the range of TAC values corresponding to healthy conditions in this work. Measurement of the TAC of human plasma (rather than in other human fluids as liquor [16]) is not yet in clinical laboratory practice, although we believe TAC will be a fundamental parameter to identify behavioral standards geared towards disease prevention and health promotion, which should be one of the main goals of the scientific research as well as in the field of investigations related to life extension.

## Figures and Tables

**Table 1:** Health conditions for blood donors assumed as reference individuals.

	Conditions
Age	18–65 y
Weight	>50 kg
Heart beats	50–100 beats/min
Blood pressure	110–180 systolic 60–100 diastolic
Health state	good
Lifestyle	no risk behavior

**Table 2:** Report of the bootstrap estimation of reference intervals for male and female subgroups.

Statistics	Males		Females	
	Upper Limit	Lower Limit	Upper Limit	Lower Limit
Median	1248	727	1048	637
Mean	1251	729	1064	641
Standard deviation	22	29	31	24
Min	1200	684	1019	571
Max	1352	791	1136	717
Percentiles				
5th	1223	690	1037	608
25th	1238	694	1044	621
50th	1248	727	1048	637
75th	1258	753	1071	644
95th	1302	778	1134	681
TAC lower limit	727 (690–778)		637 (608–681)	
TAC upper limit	1248 (1223–1302)		1048 (1037–1134)	

Note: median, mean, standard deviation, min, max, percentiles and lower and upper limit are expressed as µmol L^−1^; lower and upper limits are reported with 0.90 confidence interval.

## Data Availability

Data is contained within the article or supplementary material.

## Supplementary Materials

The following are available online at https://www.mdpi.com/2076-3921/10/1/58/s1, Figure S1: Box-and-whisker plot of male subgroup, Figure S2: Box-and-whisker plot of female subgroup, Table S1: D’Agostino–Pearson normality test, Table S2: Descriptive statistic for healthy and pathological subgroups, Table S3: Assessment of the age effect on the TAC values.
