# Supplementary material for: Dysmetabolisms Can Affect Total Antioxidant Capacity (TAC) of Human Plasma: Determination of Reference Intervals of TAC by Way of CUPRAC-BCS Method
Source: Antioxidants (Basel). 2021 Jan 5;10(1):58. doi: 10.3390/antiox10010058 (PMC7824953; doi:10.3390/antiox10010058)
Supplement: Supplementary file 1 [file antioxidants-10-00058-s001.pdf]

antioxidants

Supplementary Materials

Dysmetabolisms can affect Total Antioxidant Capacity (TAC) of human plasma: determination of reference intervals of TAC by way of CUPRAC-BCS method

Enrico Prenesti [1], Silvia Berto [1*], Fabio Gosmaro [1, 2], Marco Bagnati [3] and Giorgio Bellomo [3]

[1] Department of Chemistry, University of Turin, Via Pietro Giuria 5, 10125, Turin and Italy; enrico.prenesti@unito.it (E.P.); silvia.berto@unito.it (S.B.)
[2] Istituto professionale di Stato per servizi alberghieri e ristorazione G. Giolitti, piazza IV Novembre, 12080, Mondovì (CN), Italy; fabiogos@yahoo.it (F.G.)
[3] Major of the Charity Hospital, University of Eastern Piedmont Amedeo Avogadro, C.so Giuseppe Mazzini, 18, 28100,Novara, Italy; marco.bagnati@maggioreosp.novara.it (M.B.); bellomo.giorgio@gmail.com (G.B.)
* Correspondence: silvia.berto@unito.it; Tel.: +39-011-670-5279 (S.B.)

Preliminary statistical treatment

Table S1. D'Agostino-Pearson normality test.

Statistics[a]	Male	Female
**n**	261	152
**g1**	-0.209	0.073
**g2**	0.114	-0.100
**Z_1(g1)**	-0.846	0.290
**Z_2(g2)**	0.505	0.418
**K^2**	0.971	0.259
**Degree of freedom**	2	2
**p-value**	0.615	0.878

[a] *n* is the number of observations, g1 is the sample skewness, g2 is the sample kurtosis, Z_1(g1) is the standard normal deviate of sample skewness, Z_2(g2) is the standard normal deviate of sample kurtosis and $K^2 = (Z_1(g1))^2 + (Z_2(g2))^2$ is the statistic criterion and if the null hypothesis of normality is true, then K^2 is approximately χ^2-distributed with 2 degrees of freedom.

Being both p-value > 0.05, it was possible to confirm the Gaussian distribution of the two subgroups.

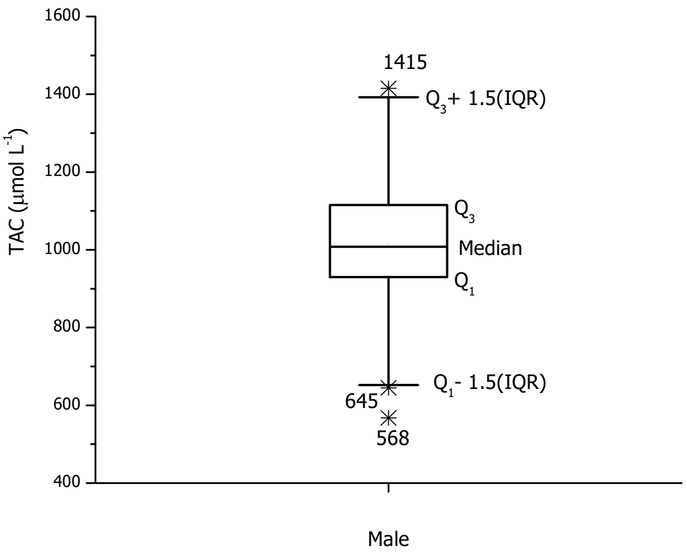

Figure S1. Box-and-whisker plot of male subgroup for the inspection of the outliers (plotted with an asterisk).

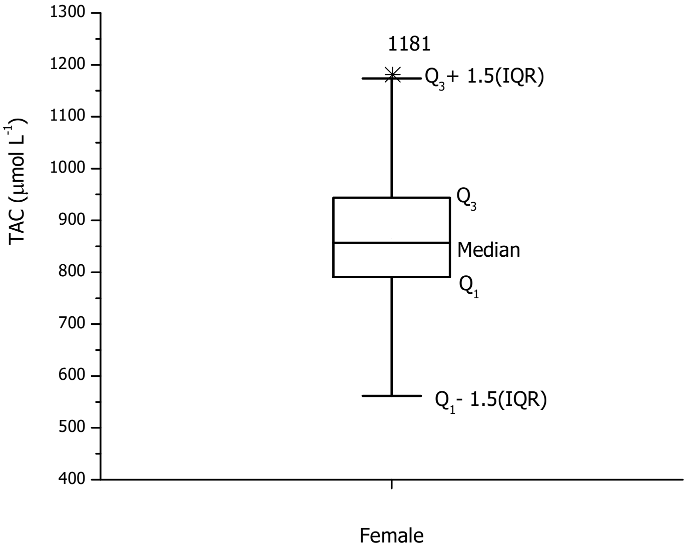

Figure S2. Box-and-whisker plot of female subgroup for the inspection of the outliers (plotted with an asterisk).

Table S2. Descriptive statistic (TAC values, μmol L^{-1}) for healthy and pathological subgroups.

Subgroup	n	Median	Mean	St. dev.	Min	Max	25 percentiles	75 percentiles	Age[a]
	**Descriptive statistic on original dataset of healthy donors (n = 413)**								
**Male**	261	1008	1015	143	568	1415	929	1115	19 – 65
**Female**	152	856	864	115	571	1181	787	944	19 – 62
	**Descriptive statistic on dataset of healthy donors with outlier removal (n = 409)**								
**Male**	258	1008	1016	137	679	1379	930	1115	19 – 65
**Female**	151	856	862	113	571	1136	786	944	19 – 62
	**Descriptive statistic on for hyperuricemic patients**								
**Male**	54	1275	1288	91	1057	1555	1239	1333	-
	**Descriptive statistic on for hyperbilirubinemic patients**								
**Male**	25	1260	1389	452	857	2408	1121	1443	-
	**Descriptive statistic on for dialysis patients**								
**Pre-dialysis**	50	1485	1520	344	727	2722	1382	1610	-
**Post-dialysis**	50	728	737	109	527	1047	659	818	-
	**Descriptive statistic on for diabetic patients**								
**Male**	55	1020	1033	138	748	1296	927	1137	-
**Female**	38	957	973	113	750	1171	915	1054	-

[a] age expressed in years.

Table S3. Assessment of the age effect on the TAC values. Statistical parameters of male and female subgroups data subdivided into four age-intervals and the corresponding one-way ANOVA reports.

	**Males**				
**Age interval** [a]	**18 – 30**	**31 – 40**	**41 – 50**	**51 – 65**	
**n** [b]	46	98	72	42	
**Mean**	998	1030	998	1037	
**Median**	984	1048	991	1015	
**Standard deviation**	139	134	136	142	
**Min**	684	690	679	780	
**Max**	1379	1328	1255	1370	
	**ANOVA report (α =0.05)**				
	**Sum of squares**	**Degrees of freedom**	**Variance**	**F**	**p-value**
**Age intervals**	$76.1 \cdot 10^3$	3	$25.4 \cdot 10^3$	1.3643	0.2530
**Prediction error**	$47.3 \cdot 10^5$	254	$18.6 \cdot 10^3$		
	**Females**				
**Age interval** [a]	**18 – 30**	**31 – 40**	**41 – 50**	**51 – 65**	
**n** [b]	45	44	45	17	
**Mean**	847	875	844	871	
**Median**	829	885	861	875	
**Standard deviation**	115	113	112	114	
**Min**	637	600	571	683	
**Max**	1113	1136	1044	1134	
	**ANOVA report (α =0.05)**				
	**Sum of squares**	**Degrees of freedom**	**Variance**	**F**	**p-value**
**Age intervals**	$19.2 \cdot 10^3$	3	$64.1 \cdot 10^2$	0.4968	0.6894
**Prediction error**	$19.0 \cdot 10^5$	147	$12.9 \cdot 10^3$		

[a] age interval expressed in years.

[b] *n* is the number of observations; mean, median, standard deviation, min and max are expressed in μmol L^{-1}.
